# Dietary *Yucca schidigera* and *Bacillus subtilis* enhance growth and immunity while reducing ammonia discharge in Nile tilapia (*Oreochromis niloticus*)

**DOI:** 10.1007/s10695-025-01607-7

**Published:** 2025-12-23

**Authors:** Doaa M. Elsisy, Heba M. Abdel-Ghany, Amr mounir Helal, Sara O. Makled, Mamdouh Ali Alharbi, Mohamed El. Salem

**Affiliations:** 1 National Institute of Oceanography and Fisheries (NIOF), Cairo, Egypt; 2 Faculty of Science, Alexandria University, Alexandria, Egypt; 3 Faculty of Marine Sciences, King Abdulaziz University, Jeddah, Saudi Arabia

**Keywords:** Ammonia, *Bacillus*, Growth performance, Immunostimulants, Nile tilapia, Yucca

## Abstract

This study aimed to evaluate the effects of dietary inclusion of *Yucca schidigera* extract and *Bacillus subtilis* on the growth performance, immune status, and water quality of Nile tilapia (*Oreochromis niloticus*) fingerlings. A total of 120 fish (13.54 ± 0.32 g) were randomly distributed into four groups (three replicates each). Each group fed one of the four experimental diets for 65 days: a control diet without additives (CON), a yucca-supplemented diet (0.2 g kg⁻^1^; Y), a *Bacillus*-supplemented diet (1.0 g kg⁻^1^; B), or a combined yucca and *Bacillus* diet (0.2 + 1.0 g kg⁻^1^; YB). Water quality analysis indicated that unionized ammonia (NH₃) concentration was the highest in CON and the lowest in YB (*p* ≤ *0.05*). B and YB exhibited superior growth performance and feed efficiency compared to Y and CON (*p* ≤ *0.05*). The highest activities of protease, amylase, and lipase were recorded in YB, followed by B and then Y, while CON exhibited the poorest results. Phagocytic activity, respiratory burst, and complement activity were the greatest in YB, intermediate in B and Y, and the lowest in CON (*p* ≤ *0.05*). Lysozyme activity was found to be significantly higher in B, followed by YB, with the lowest activity observed in CON (*p* ≤ *0.05*). Antioxidant enzyme levels (superoxide dismutase, glutathione peroxidase, reduced glutathione, and total antioxidant capacity) were maximized in YB and minimized in CON with intermediate results in B (*p* ≤ *0.05*), whereas malondialdehyde concentration showed the opposite trend. YB and B exhibited higher levels of *interleukin-8* and *tumor necrosis factor alpha* compared to Y, while CON showed the lowest expression (*p* ≤ *0.05*). *Interleukin-1 beta* expression was the highest in B, followed by YB, then Y, with the lowest levels observed in CON (*p* ≤ *0.05*). Thus, dietary supplementation with *Yucca schidigera* extract and *Bacillus subtilis*, in combination, may represent an effective strategy to improve water quality, growth performance, and immune-antioxidant responses in Nile tilapia.

## Introduction

The Nile tilapia (*Oreochromis niloticus*) ranks second to carp in importance as a farmed fish, with a global production reaching approximately 5.5 million tonnes in 2023 (FAO 2025). Currently, more than 120 countries worldwide engage in tilapia production (FAO 2025). Currently, intensive and semi-intensive aquaculture are the dominant fish farming methods applied. It is well established that intensive aquaculture systems produce substantial ammonia, a primary metabolic waste from fish and breakdown of feed residues, leading to increased organic matter accumulation at the pond bottom and resulting in decreased dissolved oxygen levels (Sriyasak et al. 2015).

Consequently, high ammonia concentration in fish rearing water leads to elevated ammonia levels in fish plasma, as it is absorbed through the gill epithelium (Hegazi and Hasanein 2010). Ammonia is found in water in two forms: NH₄⁺ (ionized ammonium) and NH₃ (unionized ammonia) (Edwards et al. 2024). Together, they are called total ammonia nitrogen (TAN) (Edwards et al. 2024). Unionized ammonia is considered much more hazardous than ammonium ions and has adverse impacts on the metabolism, growth, and immunology of aquatic species (Randall and Tsui 2002; Reddy-Lopata et al. 2006; Maas et al. 2012).

Accumulation of high NH_3_ concentrations in fish rearing water can cause oxidative stress, respiratory and metabolic disorders, gill damage, apoptosis of the hepatopancreas, endoplasmic reticulum stress, neurotoxicity, muscular depolarization, changes in mucus production, reduced growth rate, and even mortality (Lang et al. 1987; Atwood et al. 2000; Smart 2006; Maas et al. 2012; Liang et al. 2016; Ming et al. 2017; Luo et al. 2022). Furthermore, long-term exposure to ammonia in fish can change the expression of genes relevant to the immune system and antioxidant enzymes (Qi et al. 2017). Thus, ammonia toxicity may restrict development in intensive aquaculture (Adhikari 1999). Identifying methods to alleviate the impacts of ammonia stress is currently a crucial priority for aquaculturists. This could be accomplished by incorporating natural and eco-friendly additives into fish diets. These additives may help reduce ammonia levels or mitigate its harmful effects by boosting immunity and enhancing antioxidant capacities.

Plant extracts show potential as a natural additive in aquaculture, and *Yucca schidigera, in particular,* has shown efficacy in mitigating excessive ammonia generation from aquatic species (Adegbeye et al. 2019; Tidwell et al. 1992). For example, a significant decrease in total ammonia nitrogen concentration and unionized ammonia was recorded after adding *Yucca schidigera* extract to the rearing water of Nile tilapia (*O. niloticus*) (Mohamed et al. 2025). This was accompanied by an increase in dissolved oxygen levels and a decrease in water pH (Mohamed et al. 2025). Yucca mitigates ammonia levels by regulating pH and promoting nitrifying bacteria that transform toxic ammonia into less toxic nitrites (Gudipati 2025). Furthermore, yucca possesses anti-inflammatory, antioxidant, and growth-promoting properties; its active constituents, steroidal saponins and polyphenolics, function as antioxidants and free radical scavengers that may mitigate reactive oxygen species, which trigger inflammatory responses (Ayasan et al. 2005; Gudipati 2025). Thus, yucca may serve as an effective agent for reducing ammonia discharge in fish water.

The addition of probiotics, which are beneficial bacteria that can boost the immune system, improve growth performance, and change the flora in the intestines, has been approved (Kuebutornye et al. 2020; Olmos et al. 2020; Soltani et al. 2019). In this context, *Bacillus species* are gram-positive, rod-shaped bacteria recognized as beneficial microorganisms. Particularly, *Bacillus subtilis* produces various growth-promoting agents, digestive enzymes, and antimicrobial compounds (Alcaraz et al. 2010). Numerous studies have investigated the effects of using *Bacillus species* in aquaculture practices, focusing on their impact on metabolism and immune responses (Ahmadifar et al. 2019; Shi et al. 2020; Olmos et al. 2020; Sadeghi et al. 2020), as well as their role in maintaining water quality (Hlordzi et al. 2020). Adding *Bacillus* spp. mixture at a concentration of 0.2 or 0.5 g kg⁻^1^ into the diets of Nile tilapia (*Oreochromis niloticus*) for 90 days reduced water ammonia levels compared with the control group (Atef et al. 2024).

Despite intensive investigations conducted on *Yucca schidigera* and *Bacillus subtilis* separately, their synergistic effects on Nile tilapia (*O. niloticus*) have yet to be explored. Therefore, the aim of this study is to evaluate how the use of yucca and *Bacillus* bacteria influences the growth performance, immune status, and water quality of cultured Nile tilapia.

## Materials and methods

### Experimental diets

The chemical analysis and composition of the experimental diets are shown in Table 1. Four diets that were isocaloric (445 kcal 100 g^-1^ DM) and isonitrogenous (280 g kg^-1^ protein) were formulated. Yucca extract (0.2 g kg⁻^1^; Y), *Bacillus subtilis* (1 g kg⁻^1^; B) (each gram of *B. subtilis* contains 1.0 × 10^7^ CFU), or a combination of yucca extract and *B. subtilis* (0.2 and 1 g kg⁻^1^, respectively; YB) were added to three different diets, while the control diet (CON) was kept unaltered. *Yucca schidigera* and *Bacillus subtilis* doses were chosen based on previous studies (Deyab et al. 2004; Srisapoome et al. 2017; Zhang et al. 2024). The dry and ground components were thoroughly mixed by hand as the oils were poured. Subsequently, warm water was slowly added to combine all the components. The pellets were produced from the dough using a meat grinder with an appropriate diameter. Diets were dried at 60°C in an electric oven for 24 h and thereafter kept at − 20°C in plastic containers.

**Table 1:** Formulation and proximate biochemical composition of the experimental diets fed to Nile tilapia (*O. niloticus*)

**Ingredients** (g kg^−1^)	**Diet 1 (CON)**	**Diet 2 (Y)**	**Diet 3 (B)**	**Diet 4 (YB)**
Fish meal (700 g kg^−1^ CP)	50.0	50.0	50.0	50.0
Soybean meal (440 g kg^−1^ CP)	490.0	490.0	490.0	490.0
Corn flour	380.0	380.0	380.0	380.0
Minerals and vitamins^1^	20.0	20.0	20.0	20.0
Yucca	0.0	0.2	0.0	0.2
*Bacillus subtilis*	0.0	0.0	1.0	1.0
Starch	10.0	9.8	9.0	8.8
Oils (50% fish oil:50% corn oil)	40.0	40.0	40.0	40.0
Di-calcium phosphate	10.0	10.0	10.0	10.0
**Proximate analysis %**				
Crude protein	28.07	28.19	27.88	27.81
Crude lipid	6.48	6.54	6.57	6.70
Ash	6.53	6.43	6.50	6.58
Fiber	4.35	4.48	4.41	4.52
Gross energy (kcal 100 g^−1^ DM)	443.75	444.14	443.81	443.66

^1^Vitamin and minerals mixture contains (mg kg^-1^ or IU kg^-1^ of dry vitamins and minerals powder): Vit. A 2.200.000 IU., Vit. D_3_ 1.100.000 I.U., Vit. E 1.500 I.U., Vit. K 800 mg, Vit. B_1_ 1100 mg, Vit. B_2_ 200 mg, Vit. B_6_ 2.000 mg, Vit. H 15 mg, Vit. B_12_ 4 mg, Vit. C 3.000 mg, iron 160 mg, magnesium 334 mg, copper 21.6 mg, zinc 21.6 mg, selenium 25 mg, cobalt 2.38 mg

### Fish and the experimental facilities

Nile tilapia (*Oreochromis niloticus*) fingerlings were purchased from a commercial farm in Alexandria and maintained in a one-m^3^ fiberglass tank at Al-Max Research Station, the National Institute of Oceanography and Fisheries. One hundred and twenty fish (13.54 ± 0.32 g fish^-1^) were restocked in 12 glass aquaria (70 L aquarium^-1^) at a density of 10 fish per aquarium. There were three replicates of each treatment. To facilitate adaptation to the experimental diets, fish fed the control diet for 2 weeks following their transfer to the aquaria. For the following 65 days, fish were given the experimental diets three times each day until satiety. Each aquarium received dechlorinated tap water with constant aeration. The diet quantity consumed per treatment was determined weekly by comparing the weights of the food containers before and after feeding. Feces were collected by siphoning 2 h after feeding. The study was subjected to natural light conditions.

#### Ethical declaration

The experiments were performed in accordance with guidelines outlined and approved by the National Institute of Oceanography and Fisheries Committee for Institutional Care of Aquatic Organisms and Experimental Animals (NIOF-IACUC), Egypt.

### Water quality monitoring

The water quality parameters monitored every week throughout the experiment included ionized ammonia (NH_4_^+^), unionized ammonia (NH_3_), nitrite (NO₂⁻), nitrate (NO₃⁻), dissolved oxygen (DO), temperature, and pH. All parameters were measured using the Hanna HI83303 photometer.

### Productive performance

All fish in each aquarium were collected and weighed before starting and at the end of the feeding trial. Growth, feed utilization, and other biometric indices were calculated according to the following equations (El-Sayed 2006):

$$\text{Survival rate\%}=\frac{\text{Final number of fish}}{\text{Initial number of fish}}\times 100$$

$$Feed\,intake \left(g\right)=Total\,amount\,of\,consumed\,diet$$

$$Weight\,gain \left(WG, g\right)=Final\,weight-Initial\,weight$$

$$Specific\,growth\,rate \left(SGR,\mathrm{\%}da{y}^{-1}\right)=\frac{In\left(Final\,weight\left(g\right)\right)-In\left(Initial\,weight\left(g\right)\right)}{Trial\,duration}\times 100$$

$$Average\,daily\,gain \left(ADG,g da{y}^{-1}\right)=\frac{Final\,weight\left(g\right)-initial\,weight\left(g\right)}{Trial\,duration}$$

$$Feed\,conversion\,ratio (FCR) =\frac{Total\,dry\,feed\,intake (g) }{ Total\,fish\,weight\,gain (g)}$$

$$Protein\,efficiency\,ratio\,(PER) =\frac{WG (g) }{ Total\,dry\,protein\,intake (g)}$$

$$Protein\,productive\,value\,(PPV) =\frac{Protein\,gain\,(g\,wet\,weight) }{Protein\,fed\,on\,dry\,weight\,basis\,(g)}\times 100$$

### Body biochemical composition

Prior to the start of the feeding experiment, 10 fish were randomly picked and frozen at − 20°C for the whole-body biochemistry examination. Six fish from each treatment were collected at the termination of the feeding experiment for the whole-body biochemical examination. Fish were sedated with 0.1 mL L^⁻1^ clove oil (Fernandes et al. 2017), the crude protein, lipid, moisture, and ash contents of all samples were determined using methods outlined by AOAC (2009).

### Analysis of digestive enzymes

After the feeding experiment, six fish were randomly selected from each treatment. Fish were anesthetized with 0.1 mL L⁻^1^ clove oil (Fernandes et al. 2017), dissected under aseptic conditions, and their intestines were carefully excised. The intestinal contents were collected, washed, homogenized, and centrifuged at 5000 × g for 30 min at 4°C. The supernatant was collected and stored at 4°C until enzymatic analyses, which were performed within 24 h of extraction. The activities of lipase, amylase, and protease were determined following the procedures described by Sun et al. (2011) and expressed as U mg⁻^1^ protein.

### Blood sampling

After the feeding experiment, six fish were randomly selected from each treatment for blood collection and subsequent biochemical analysis. Clove oil (0.1 mL L^−1^) (Fernandes et al. 2017) was used to sedate the fish. Blood samples (1 mL) were collected from the fish caudal vein and stored in plastic microtubes without anticoagulant and centrifuged for 10 min at 3000 × g to separate the serum for the biochemical analyses.

### The immunological analyses

Phagocytic activity (PA) was measured by inoculating 200 μL of leukocyte suspensions with 100 μL of formalin-killed *Staphylococcus aureus* in phosphate-buffered saline at room temperature for 30 min. Following that, the mixture was centrifuged at 3000 × g for 5 min at 4°C. Pellets were collected, smeared, and stained with Wright-Giemsa solution (Sigma-Aldrich, St. Louis, MO, USA). Phagocytic cells were counted microscopically, following Sun et al. (2010). PA was calculated using the formula outlined below:

$$PA=100\times \frac{\text{number of phagocytes containing }S. aureus}{\text{phagocyte counted}}$$

Respiratory burst (RB) activity was determined spectrophotometrically by quantifying the formazan produced during the nitroblue tetrazolium (NBT)–O₂ redox reaction (Franco et al. 2017). One unit of RB activity was defined as a 0.001 increase in absorbance at 630 nm min^-1^.

To measure alternative complement activity (ACH50), 0.5 mL of serially diluted serum was combined with 0.2 mL of erythrocyte suspension (2 × 107 cells mL^-1^). The mixture was then incubated for 2 h at 20°C in a solution containing 10 mM EGTA and 10 mM MgCl_2_ (pH 7.0). The hemolytic process was then stopped by adding 10 mM EDTA and 1.4 mL of gelatin veronal buffer. At 414 nm, the optical density (OD) was measured. According to Cheng et al. (2007), one unit of ACH50 is equivalent to the volume of serum complement that causes 50% hemolysis.

In order to analyze the lysozyme (LYZ) activity, 50 μL of blood serum was added to 950 μL of *Micrococcus lysodeikticus* suspension (200 mg mL^−1^, Jiancheng, Nanjing, Jiangsu, China). The mixture was then mixed with 0.05 M sodium phosphate buffer (pH 6.2) at 25°C (Sun et al. 2010). OD was determined by a spectrophotometer at 530 nm. The quantity of enzyme required to produce a 0.001 min^−1^ mL^−1^ drop in absorbance is equivalent to one unit of LYZ.

### The antioxidant analyses

Liver samples (three samples per replicate) were collected to assess lipid peroxidation and antioxidant status. Approximately 1 g of liver tissue was homogenized with 10 ml of cold phosphate buffer using an electrical homogenizer surrounded by ice. The homogenate was centrifuged at 2800 × g for 30 min. Lipid peroxidation was assessed spectrophotometrically by determining malondialdehyde (MDA) content, according to the method of Ohkawa et al. (1979). Superoxide dismutase (SOD) activity was analyzed using a commercial reagent kit (Jiancheng, Nanjing, Jiangsu, China), based on the inhibition of NBT reduction by superoxide radicals. One unit of SOD activity was defined as the amount of enzyme required to produce 50% inhibition of the NBT reduction rate. OD was measured spectrophotometrically at 550 nm (Sun et al. 2010). Glutathione peroxidase (GPx) activity was determined following the method of Khan et al. (2016), with OD of NADPH measured at 340 nm and 37°C. The non-enzymatic antioxidant reduced glutathione (GSH) was quantified according to Owens and Belcher (1965) using commercial diagnostic kits (MyBioSource) following the manufacturer’s instructions. The total antioxidant capacity (TAC) was assessed using a spectrophotometer set at a wavelength of 532 nm, following the Tween 80 oxidation technique as described by Galaktionova et al. (1998).

### Cytokine gene expression

Using the Gene Jet RNA Purification Kit, the liver was separated and kept at − 70°C for subsequent RNA extraction. *Interleukin-1 beta* (*IL1b*), *interleukin-8* (*IL8*), and *tumor necrosis factor alpha* (*TNF-α*) are three pro-inflammatory immune-related genes that were analyzed in duplicate by real-time PCR (RT-PCR), as described by Makled et al. (2019) (Table 2). Using a Bio-Rad thermocycler, the reaction was carried out at 45°C for 60 min, after which it was halted by raising the temperature to 70°C. *β-actin* was used as an internal reference to normalize gene expression data. The 2^ − ΔΔCT method was used to analyze the data (Livak and Schmittgen 2001).

**Table 2:** Primer sequences used for RT-PCR analysis of cytokine gene expression in Nile tilapia (*O. niloticus*)

Gene name (symbol)	Primer sequence (5′–3′)	Accession No	Primer efficiency (%)	Reference
***Beta-actin***	F: 5′-AGACATCAGGGTGTCATGGTTGGT-3′ R: 5′-CTCAAACATGATCTGTGTCAT- 3′	M24113	98.4	(Chi et al., 2014)
***Interleukin-1 beta**** (IL-1β)*	F: 5′-ACCAGCTGGATTTGTCAGAAG- 3′ R: 5′- ACATACTGAATTGAACTTTG- 3′	AB010701	96.7	(Chi et al., 2014)
***Tumor necrosis factor-alpha**** (TNF-α)*	F: 5′- GGTGATGGTGTCGAGGAGGAA-3′ R: 5′-TGGAAAGACACCTGGCTGTA- 3′	AJ311800	97.5	(Chi et al., 2014)
***Interleukin 8**** (IL-8)*	F: 5′-GCACTGCCGCTGCATTAAG-3′ R: 5′-GCAGTGGGAGTTGGGAAGAA-3′	DQ061114.1	99.1	(Shourbela et al., 2021)

### Statistical analysis

Data were analyzed using one-way analysis of variance (ANOVA), and significant differences among means were determined by Duncan’s multiple range post hoc test. Differences were considered statistically significant at *p* ≤ *0.05*. All statistical analyses were performed using SPSS software (version 23.0, IBM Corp., Armonk, NY, USA).

## Results

### Water quality

**Fig. 1:**
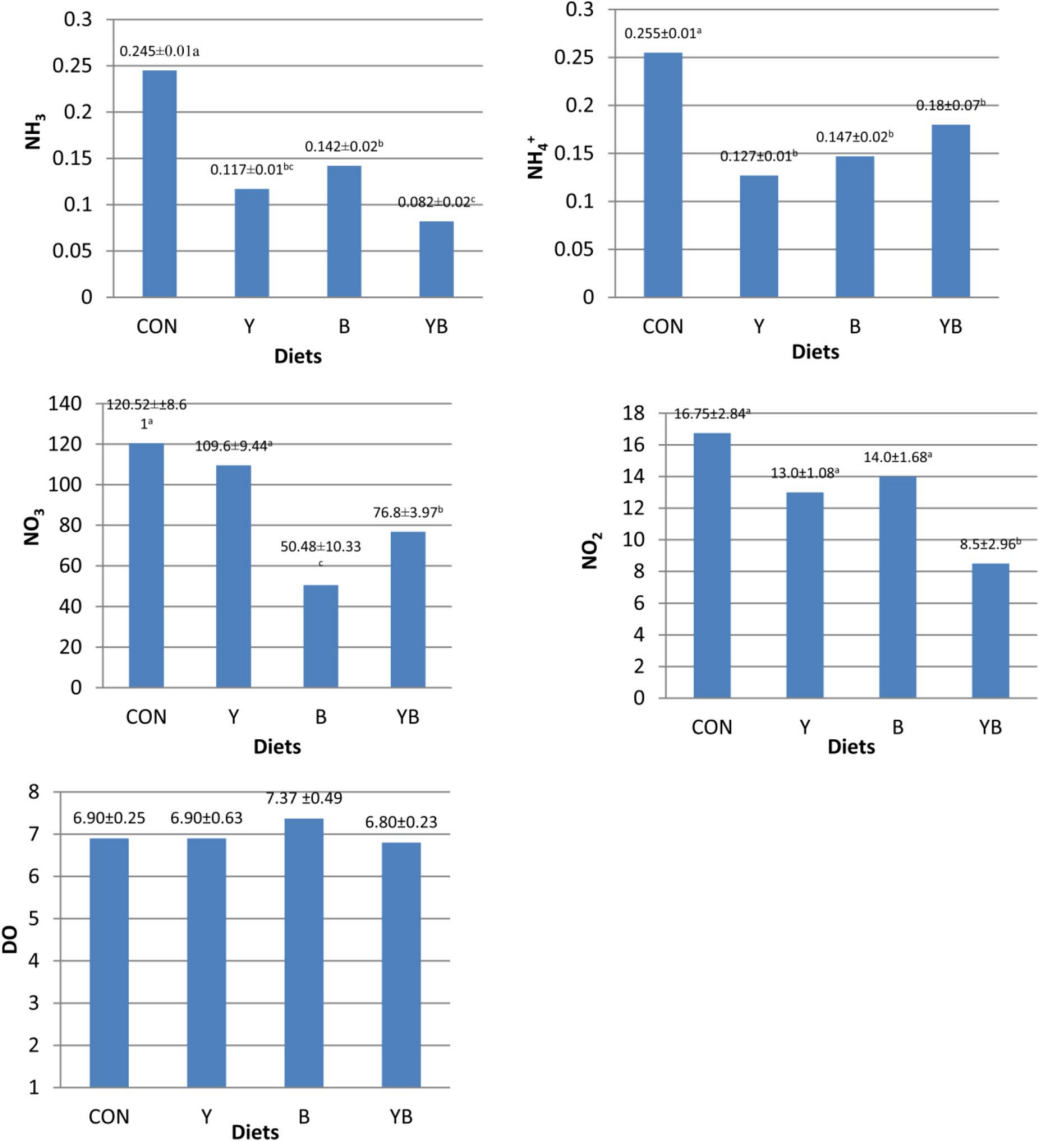
Effect of the experimental diets on water quality parameters of Nile tilapia (*Oreochromis niloticus*) rearing water, including **a** unionized ammonia (NH₃), **b** ionized ammonia (NH₄⁺), **c** nitrite (NO₂⁻), **d** nitrate (NO₃⁻), and **e** dissolved oxygen (DO). Data are presented as mean ± standard deviation (SD). Different superscript letters indicate significant differences among treatments (*p* ≤ 0.05). All values are expressed in mg L⁻^1^ (ppm)

Figure 1 shows the water quality parameters that were measured in the aquaria of Nile tilapia (*O. niloticus*). The highest concentration of NH₃ was significantly recorded in CON compared to all other groups, while Y and B displayed an intermediate reduction (*p* ≤ *0.05*). The lowest NH₃ level was recorded in YB. For NH₄⁺, CON produced higher results compared to the other groups (*p* ≤ *0.05*). YB had a much lower concentration of NO₂⁻ than the other groups (*p* ≤ *0.05*), and there were no significant differences between CON, Y, and B (*p* ≥ *0.05*). For NO₃⁻, the highest values were recorded in CON and Y, followed by YB, whereas B exhibited the lowest value (*p* ≤ *0.05*). DO levels showed no significant differences among groups (*p* ≥ *0.05*). Water temperature and pH remained constant at 25 ± 2.1°C and 7.7, respectively.

### Growth performance and feed utilization

Table 3 presents the growth performance and feed utilization results of Nile tilapia (*O. niloticus*) fed the experimental diets. B and YB had the highest final weight (FW), weight gain (WG), average daily gain (ADG), and specific growth rate (SGR) values, followed by the CON and Y (*p* ≤ *0.05*). There were no significant differences in feed intake among the various treatments (*p* ≥ *0.05*). CON and Y exhibited the highest feed conversion ratio (FCR) values, while B and YB gave the lowest (*p* ≤ *0.05*). Additionally, B and YB had higher protein efficiency ratio (PER) and protein productive value (PPV) than the Y and CON (*p* ≤ *0.05*).

**Table 3:** Growth performance and feed utilization of Nile tilapia (*O. niloticus*) fed the experimental diets

	**CON**	**Y**	**B**	**YB**
Initial weight	14.63 ± 0.12	14.80 ± 0.42	14.54 ± 0.20	14.56 ± 0.18
Final weight	27.53 ± 0.66^b^	27.20 ± 0.74^b^	30.08 ± 1.07^a^	30.05 ± 0.45^a^
Gain	12.90 ± 0.74^b^	12.40 ± 0.38^b^	15.54 ± 1.26^a^	15.49 ± 0.34^a^
ADG	0.20 ± 0.01^b^	0.19 ± 0.00^b^	0.24 ± 0.20^a^	0.24 ± 0.00^a^
SGR	0.97 ± 0.04^b^	0.94 ± 0.02^b^	1.12 ± 0.07^a^	1.11 ± 0.01^a^
Feed intake	20.41 ± 0.88	20.09 ± 0.48	20.62 ± 1.06	20.90 ± 0.33
FCR	1.58 ± 0.05^a^	1.62 ± 0.03^a^	1.33 ± 0.09^b^	1.35 ± 0.01^b^
PER	2.25 ± 0.08^b^	2.19 ± 0.06^b^	2.71 ± 0.20^a^	2.67 ± 0.06^a^
PPV	45.69 ± 2.56^b^	45.44 ± 2.50^b^	59.86 ± 4.23^a^	57.54 ± 1.32^a^

Data are presented as mean values ± standard deviation. Data in the same row with different superscripts significantly differ (*p* ≤ *0.05*). *ADG*, average daily gain; *SGR*, specific growth rate; *FCR*, feed conversion ratio; *PER*, protein efficiency ratio; *PPV*, protein productive value

### Body chemical composition

Table 4 shows the results of the chemical composition of the whole body of Nile tilapia (*O. niloticus*) fed on the experimental diets. At the end of the feeding experiment, the dry matter and ash did not differ among treatments (*p* ≥ *0.05*). The highest crude protein (CP) values were obtained in B and YB, followed by Y and CON (*p* ≤ *0.05*). The lowest crude lipid (CL) value was detected in YB and B, while CON and Y gave the highest results (*p* ≤ *0.05*); however, B was not significantly different from CON or Y (*p* ≥ *0.05*).

**Table 4:** Whole-body chemical composition (%) of Nile tilapia (*O. niloticus*) fed the experimental diets

	**CON**	**Y**	**B**	**YB**
Dry matter	33.15 ± 0.46	32.20 ± 0.55	33.26 ± 0.56	33.28 ± 0.74
Crude protein	54. 70 ± 0.90^b^	55.40 ± 0.41^b^	57.91 ± 0.47^a^	57.07 ± 1.20^a^
Crude lipid	25.54 ± 0.60^a^	26.20 ± 0.41^a^	24.42 ± 1.42^ab^	23.48 ± 1.00^b^
Ash	18.24 ± 0.94	17.50 ± 0.75	16.92 ± 0.58	17.86 ± 1.41

Data are presented as mean values ± standard deviation. Data in the same row with different superscripts significantly differ (*p* ≤ *0.05*)

### The digestive enzymes

Table 5 presents the digestive enzyme activities in Nile tilapia (*O. niloticus*) fed on the experimental diets. Protease and amylase activities increased progressively from CON to Y and B, with the highest levels recorded in YB (*p* ≤ *0.05*). Additionally, YB exhibited the highest lipase activity, while CON demonstrated the lowest activity, with Y and B showing intermediate levels (*p* ≤ *0.05*).

**Table 5:** Effect of the experimental diets on digestive enzyme activities of Nile tilapia (*O. niloticus*)

Activity (U mg^−1^ protein)	**CON**	**Y**	**B**	**YB**
Protease	51.47 ± 1.12^d^	56.00 ± 0.26^c^	62.83 ± 0.25^b^	66.87 ± 0.15^a^
Amylase	29.87 ± 0.15^d^	31.50 ± 0.70^c^	37.37 ± 0.49^b^	39.77 ± 0.55^a^
Lipase	58.00 ± 0.75^c^	68.30 ± 0.56^b^	70.97 ± 0.379^b^	77.87 ± 1.80^a^

Data are presented as mean values ± standard deviation. Data in the same row with different superscripts significantly differ (*p* ≤ 0.05)

### The immunological analyses

The immunological evaluation of Nile tilapia (*O. niloticus*) fed the experimental diets are shown in Table 6. YB showed the highest PA, RB, and ACH50 levels, significantly surpassing all other groups. This was followed by B, and then Y, with the lowest values recorded in CON (*p* ≤ *0.05*). The highest LYZ value was recorded in B, followed by YB and then Y. The lowest overall value of LYZ was found in CON (*p* ≤ *0.05*).

**Table 6:** Effect of the experimental diets on innate immunological parameters of Nile tilapia (*O. niloticus*)

	**CON**	**Y**	**B**	**YB**
PA	3.93 ± 0.47^d^	6.67 ± 0.59^c^	9.60 ± 0.36^b^	12.27 ± 0.25^a^
RB	4.10 ± 0.36^d^	4.77 ± 0.15^c^	5.53 ± 0.15^b^	7.77 ± 0.21^a^
ACH50	34.40 ± 0.62^d^	37.60 ± 0.35^c^	42.83 ± 0.21^b^	45.33 ± 0.58^a^
LYZ	82.67 ± 1.53^d^	87.00 ± 1.00^c^	114.67 ± 2.08^a^	95.00 ± 1.00^b^

Data are presented as mean values ± standard deviation. Data in the same row with different superscripts significantly differ (*p* ≤ 0.05). *PA*, phagocytic activity; *RB*, respiratory burst; *ACH50*, complement activity; *LYZ*, lysozyme

### The antioxidant analyses

The antioxidant status of Nile tilapia (*O. niloticus*) fed the experimental diets is presented in Table 7. MDA showed a progressive decrease from CON, Y and B to YB (*p* ≤ *0.05*). YB exhibited the highest levels of SOD activity, followed by Y and B, while the lowest level was recorded in CON (*p* ≤ *0.05*). GPx demonstrated a similar trend, with the highest value found in YB, followed by B, and the lowest values observed in Y and CON (*p* ≤ *0.05*). In line with this pattern, GSH and TAC were significantly elevated in a progressive manner from CON, Y and B to YB (*p* ≤ *0.05*).

**Table 7:** Effect of the experimental diets on antioxidant parameters of Nile tilapia (*O. niloticus*)

Antioxidants activity	CON	Y	B	YB
MDA (U mg^−1^)	4.07 ± 0.15^a^	3.27 ± 0.15^b^	2.40 ± 0.10^c^	1.03 ± 0.15^d^
SOD (U mg^−1^)	16.63 ± 0.55^c^	24.17 ± 0.25^b^	25.30 ± 0.66^b^	31.63 ± 1.19^a^
GPx (μmol mg^−1^)	0.230 ± 0.03^c^	0.33 ± 0.02^c^	0.63 ± 0.06^b^	1.77 ± 0.15^a^
GSH (nmol g⁻^1)^	0.184 ± 0.001^d^	0.333 ± 0.001^c^	0.764 ± 0.002^b^	1.823 ± 0.002^a^
TAC (μmol mg^−1)^	0.25 ± 0.04^d^	0.60 ± 0.10^c^	1.33 ± 0.15^b^	2.27 ± 0.14^a^

Data are presented as mean values ± standard deviation. Data in the same row with different superscripts significantly differ (*p* ≤ 0.05). *MDA*, malondialdehyde; *SOD*, superoxide dismutase; *GPx*, glutathione peroxidase; *GSH*, reduced glutathione; *TAC*, total antioxidant capacity

### Cytokine gene expression

Table 8 presents the effect of the experimental diets on relative mRNA expression levels in the liver of Nile tilapia (*O. niloticus*). The highest levels of *IL-8* and *TNF-α* cytokines were recorded in B and YB, followed by Y, while CON exhibited the lowest levels (*p* ≤ *0.05*). B exhibited the highest *IL-1b* cytokine activity, followed by YB, with Y showing lower activity, while CON had the lowest activity (*p* ≤ *0.05*).

**Table 8:** Relative mRNA expression of cytokine genes in the liver of Nile tilapia (*O. niloticus*) fed the experimental diets

	CON	Y	B	YB
*IL-8*	1.20 ± 0.17^c^	6.43 ± 0.05^b^	8.23 ± 0.21^a^	7.93 ± 0.15^a^
*IL-1b*	1.17 ± 0.15^d^	2.85 ± 0.05^c^	4.58 ± 0.07^a^	3.14 ± 0.05^b^
*TNF-α*	0.53 ± 0.03^c^	2.67 ± 0.15^b^	3.48 ± 0.030^a^	3.69 ± 0.09^a^

Data are presented as mean values ± standard deviation. Data in the same row with different superscripts significantly differ (p ≤ 0.05). *IL-8*, interleukin 8; *IL-1β*, interleukin 1 beta; *TNF-α*, tumor necrosis factor alpha

## Discussion

The present study demonstrates that dietary supplementation with *Yucca schidigera* extract and *Bacillus subtilis*, individually and in combination, significantly improves the growth performance, immune status, and water quality of Nile tilapia (*O. niloticus*). Regarding water quality, the combination of yucca extract and *Bacillus* in the diet of Nile tilapia (*O. niloticus*) did not affect the mean dissolved oxygen values. This effect is likely due to the controlled experimental aeration conditions, with all aquaria having a constant air supply. The rearing water of fish that were fed dietary yucca, *Bacillus*, separately or in or a combination, showed lower ammonia (NH₃) levels compared to the control group. Notably, the yucca treatment was more effective in reducing NH_3_ levels in aquarium water. This confirms the role of yucca extract in reducing NH_3_ levels, and this reduction might be owed to some components of the yucca extract that can bind with NH_3_ (Cheok et al. 2014). These components include two active ingredients: saponin (the steroidal portion), which has surface-active properties, and glycocomponent, which is capable of adsorbing NH_3_ (Johnson et al. 1986; Cheok et al. 2014). These findings are supported by some previous studies, which suggested that both saponins (Cheeke, 1996; Makkar et al. 1999) and glycocomponents/glycoproteins (Headon et al. 1991; Wallace et al. 1994) in yucca extract may be the principal reasons for reducing NH_3_. In addition, Yucca may mitigate NH_3_ levels by functioning as a pH regulator and promoting nitrifying bacteria that transform toxic NH_3_ into less toxic nitrites (Gudipati 2025). Several studies revealed the potential of yucca extract in reducing ammonia levels in freshwater and marine fishes rearing water (Santacruz-Reyes and Chien 2012; Fayed et al. 2019). For example, a feeding trial on striped catfish (*Pangasianodon hypophthalmus*) assessed the effects of various levels of *Yucca schidigera* on total ammonia nitrogen (TAN) excretion. The results showed that fish fed yucca-supplemented diets had lower TAN levels than the control group (Güroy et al. 2012). Similarly, Mohamed et al. (2025) found that the addition of yucca extract to the rearing water of Nile tilapia (*O. niloticus*) significantly decreased the concentration of TAN and NH_3_ in wastewater discharge. Moreover, Castillo-Vargasmachucaa et al. (2015) showed that the addition of yucca extract to a recirculating system containing Pacific red snapper (*Lutjanus peru*) led to decreasing NH_3_ levels. Thus, *Yucca schidigera* played a positive role in this study by reducing NH_3_ levels in the rearing water of Nile tilapia (*O. niloticus*).

In this study, *Bacillus subtilis* also played a role in lowering NH₃ levels in water by utilizing nitrogenous compounds as nutrients. Furthermore, the administration of *Bacillus* might boost the presence of nitrifying bacteria, which have the ability to convert NH_3_ to NO₂⁻ and, therefore, to NO_3_ NO₃⁻ In support, *Bacillus subtilis* reduced the concentrations of TAN, NO₂⁻, and NO₃⁻ in Nile tilapia (*O. niloticus*) rearing water under the biofloc system (Mohammadi et al. 2020). Similarly, John et al. (2020) used *Bacillus cereus*,* Bacillus amyloliquefaciens*, and *Pseudomonas stutzeri* as bioaugmentation agents in *Oreochromis mossambicus* fingerling tanks. They found that this microbial community effectively diminished NH_3_, NO₂⁻, and NO₃⁻ in rearing water. Wang et al. (2005) mentioned that adding commercial probiotics to shrimp ponds increased the nitrifying bacteria *Nitrosomonas* and *Nitrobacter*. Moreover, Wang (2007) found that adding commercial probiotics to shrimp (*Penaeus vannamei*) ponds reduced NH_3_ and phosphate levels in the water. Consistently, Song et al. (2011) reported significant decreases in NO₂⁻, TAN, and sulfide metabolites after adding *Bacillus* sp. to shrimp pond water. Khademzade et al. (2020) revealed that adding *Bacillus cereus* and *Pediococcus acidilactici* probiotics to whiteleg shrimp (*Penaeus vannamei*) pond water considerably reduced nitrogenous compounds. Thus, the enhancement of water quality by application of probiotics can be linked to various factors, including an increase in heterotrophic and nitrifying bacteria, as well as a decrease in the breakdown of organic compounds (Miranda-Baeza et al. 2020).

Among all treatments, YB produced the lowest results of NH_3_ in the current study, indicating a synergistic effect between yucca and *Bacillus* that resulted in the maximum reduction rate of ammonia in the fish rearing water. Further research could investigate the mechanisms underlying this interaction and its potential applications in aquaculture.

In the present study, the inclusion of *Bacillus subtilis* in the *O. niloticus* diets significantly enhanced growth performance and feed utilization. Similarly, Tachibana et al. (2020) reported that incorporating both *Bacillus subtilis* and *Bacillus licheniformis* in Nile tilapia diets resulted in greater weight gain compared to the control group. This improvement in growth performance is likely attributed to the observed increase in digestive enzyme activities (lipase, amylase, and protease) in all groups fed on *Bacillus*-supplemented diets. Previous studies have confirmed that *Bacillus* enhances the activity of digestive enzymes in fish, thereby improving feed utilization and promoting growth (Hura et al. 2018; Adorian et al. 2019).

This effect may occur directly through *Bacillus*-produced exogenous enzymes or indirectly by modulating gut microbiota and supporting intestinal physiology (Soto 2017). Consequently, supplementing fish diets with certain *Bacillus* species often results in higher growth rates (Soltani et al. 2019). For instance, *O. niloticus* fed *B. subtilis* diets showed increased final body weight, weight gain, and specific growth rate, along with enhanced protease and amylase activities and reduced feed conversion ratios (Liu et al. 2017). The authors attributed these improvements to the *B. subtilis* ability to secrete exogenous enzymes such as proteases and amylases. Similarly, *O. niloticus* fed *B. subtilis* exhibited improved growth performance and digestive enzyme activity (Mohammadi et al. 2020). Supporting evidence from Wu et al. (2012) showed that probiotic *B. subtilis* also enhanced the growth of grass carp (*Ctenopharyngodon idella*) through increased digestive enzyme activities. Thus, enhanced enzyme activity in the digestive system enables Nile tilapia to more efficiently digest their diet and achieve optimal growth. Moreover, probiotic cells may serve as a direct nutrient source, providing vitamins, fatty acids, and other exogenous nutrients to the host (Promthale et al. 2019). In addition, the biochemical composition observed in this study suggests that *Bacillus* may promote protein retention and modulate lipid metabolism. These findings are consistent with previous studies on aquatic organisms (Atef et al. 2024; Allah et al. 2025; Li et al. 2025). Overall, these results collectively emphasize the role of *Bacillus* in improving growth performance, feed utilization, and body composition in Nile tilapia.

In the present study, dietary *B. subtilis* enhanced the antioxidant activity of *O. niloticus* more effectively than dietary yucca, although the inclusion of yucca was still superior to the control group. Thus, *B. subtilis* may protect against oxidative stress by significantly increasing SOD, GPx, GSH, and TAC concentrations while decreasing MDA concentration. This enhancement was achieved through *Bacillus species*, which preserve host antioxidant enzymes by producing reactive oxygen species (ROS)-scavenging exopolysaccharides or antioxidant enzymes (Kuebutornye et al. 2020). In support, diet supplementation with *B. subtilis* at specific doses successfully shields tilapia (*O. niloticus*) against oxidative stress by elevating the serum levels of SOD and TAC and lowering MDA (Mohammadi et al. 2020). In similar, tilapia-fed *Bacillus* spp. showed greater tolerance to oxidative stress in serum, skin mucus, and intestines (Kuebutornye et al. 2020). Additionally, yucca has an antioxidant effect because of its capacity to scavenge secondary reactive radicals (Cheeke et al., 2006). Moreover, the polyphenols and saponins in yucca act as organic antioxidants (Piacente et al. 2005). Through this mechanism, yucca may be able to inhibit the production of superoxide and hydrogen peroxide during regular metabolic activity (Cheeke et al., 2006). Angeles et al. (2017) found that dietary yucca mediated antioxidative ability in Nile tilapia (*O. niloticus*) subjected to hypoxic stress. According to Elbialy et al. (2020), adding yucca extract to the rearing water of Nile tilapia (*O. niloticus*) raised the hepatic activities of SOD and GPx enzymes and non-enzymatic GSH concentration while lowering the hepatic MDA content. These studies are in agreement with Wang et al. (2020), who found that mirror carp (*Cyprinus carpio*) fed yucca and subjected to NH_3_ toxicity exhibited enhanced antioxidative status. The combination of yucca and *Bacillus subtilis* in the current study resulted in the maximum antioxidant activity rather than being included separately. This conclusion confirms the synergistic effect of yucca with *Bacillus subtilis*. This finding is supported by Abdo et al. (2022), who revealed a synergistic effect between yucca and *Bacillus* when fed to Nile tilapia (*O. niloticus*).

Probiotics have well-established positive effects on animal immunity. They have been revealed to enhance fish innate immunity and phagocytosis while also producing bacteriocins that increase fish resistance to infections (Dawood et al. 2018). Following the same pattern, the present study reported that RB, PA, ACH50, and LSZ showed higher activities in YB compared to the other groups containing yucca or *Bacillus* separately, which emphasizes the synergistic effect between yucca and *Bacilluss*. However, *Bacillus* had a superior role over yucca in enhancing these parameters (RB, PA, ACH50, and LSZ), keeping the yucca treatment better than the control group. In support, *Bacillus subtilis* enhanced the Nile tilapia (*O. niloticus*) under the biofloc system in terms of immune responses (total protein, albumin, globulin, lysozyme, alternative complement, protease, immunoglobulins, alkaline phosphatase, and respiratory burst) and skin mucus parameters (total protein, lysozyme, alternative complement, protease, immunoglobulins, alkaline phosphatase, and respiratory burst) (Mohammadi et al. 2020). Tilapia (*O. niloticus*) fed *Bacillus* spp. showed similar improvements in LSZ, ACH50, and RB (Selim & Reda, 2015; Telli et al., 2014). Similarly, by binding to specific receptors on cell membranes, cytokines significantly contribute to both innate and adaptive immune responses (Secombes et al. 2001). The present study found that the inclusion of *Bacillus* or a yucca-*Bacillus* mixture in the diets of *o. niloticus *upregulated the expression levels of cytokine genes, including *IL-8*,* IL-1β*, and *TNF-α*. Du et al. (2021) found similar results where dietary supplementation of *Bacillus* enhanced LYZ activities and the expression of pro-inflammatory responses of largemouth bass (*Micropterus salmoides*), as indicated by increased mRNA levels of *IL-1β*, *IL-8*, and *TNF-α*. This effect may be attributed to the interaction of probiotics and their cellular components with the immune system through microbe-associated molecular patterns (Kuebutornye et al. 2020). Specific receptors, such as Toll-like receptors, recognize these patterns. The interaction takes place at gut-associated lymphoid tissue, stimulating immune cells and triggering the release of cytokines, which ultimately enhances immune responses (Kuebutornye et al. 2020).

In conclusion, the current study demonstrates that dietary supplementation with yucca and *Bacillus*, either separately or in combination, significantly enhances water quality, growth performance, antioxidant capacity, and immunological competence in Nile tilapia (*O. niloticus*). However, the combined supplementation produced the most significant effects, indicating a synergistic interaction between yucca and *Bacillus*. These results highlight the possibility of using *Yucca schidigera* and *Bacillus subtilis* as environmentally sustainable feed additives to improve fish health and maximize the sustainability of aquaculture systems.

## Data Availability

All data generated or analyzed during this study are included in this published article.
